# Perinephric Abscess Causing Mycotic Celiac and Splenic Artery Aneurysm: A Case Report

**DOI:** 10.7759/cureus.4988

**Published:** 2019-06-24

**Authors:** Ehizogie Edigin, Sanjay A Patel

**Affiliations:** 1 Internal Medicine, John H. Stroger, Jr. Hospital of Cook County, Chicago, USA

**Keywords:** xanthogranulomatous pyelonephritis, emphysematous pyelitis, flank abscess, mycotic aneurysm

## Abstract

Perinephric abscess is a known complication of urologic infection, sometimes requiring surgical debridement. Extension into adjacent structures is rarely reported. We present a case of a woman with xanthogranulomatous emphysematous pyelonephritis accompanied by massive perinephric abscess, resulting in celiac and splenic artery mycotic aneurysms via direct invasion.

## Introduction

Perinephric abscess is a suppurative infection between the renal cortex and Gerota’s fascia. It is often attributed to gram-negative bacilli, particularly Escherichia coli and Klebsiella pneumonia, but may also be polymicrobial in nature [1-3]. Though often an extension of adjacent renal infection, hematogenous spread of infection may also occur, particularly with Staphylococcus aureus [3,4]. The presentation is typically marked by flank pain, fever, and constitutional symptoms (i.e., fatigue, chills, and weight loss). Dysuria, urinary frequency and other lower urinary tract symptoms are often not reported due to the indolent nature of abscess development [2,5].

If not properly treated, perinephric abscess results in significant morbidity and mortality [1,5-7]. Percutaneous tube-directed drainage and antibiotic therapy are often the cornerstone of treatment, though surgical debridement and nephrectomy are essential in certain scenarios [6,8-11]. Local and distal complications from inflammation of adjacent structures, especially intraperitoneal spread, are rare [1,4,5]. There are few reported cases of mycotic aneurysm formation secondary to perinephric abscesses [12].

## Case presentation

A 57-year-old woman presented to the emergency room with one week of left-sided flank pain, flank swelling, poor appetite and subjective fever. Her medical history was significant for a remote left kidney infection five years prior requiring antibiotics and percutaneous drainage. She had not seen a physician since and had no other past medical history. On arrival to the emergency room, her blood pressure was 116/65 mm/Hg, heart rate 101 beats per minute and temperature 99.1°F. A soft, tender swelling was noted in the left flank without crepitus or erythema. The remainder of her systemic examination was unremarkable.

**Figure 1:**
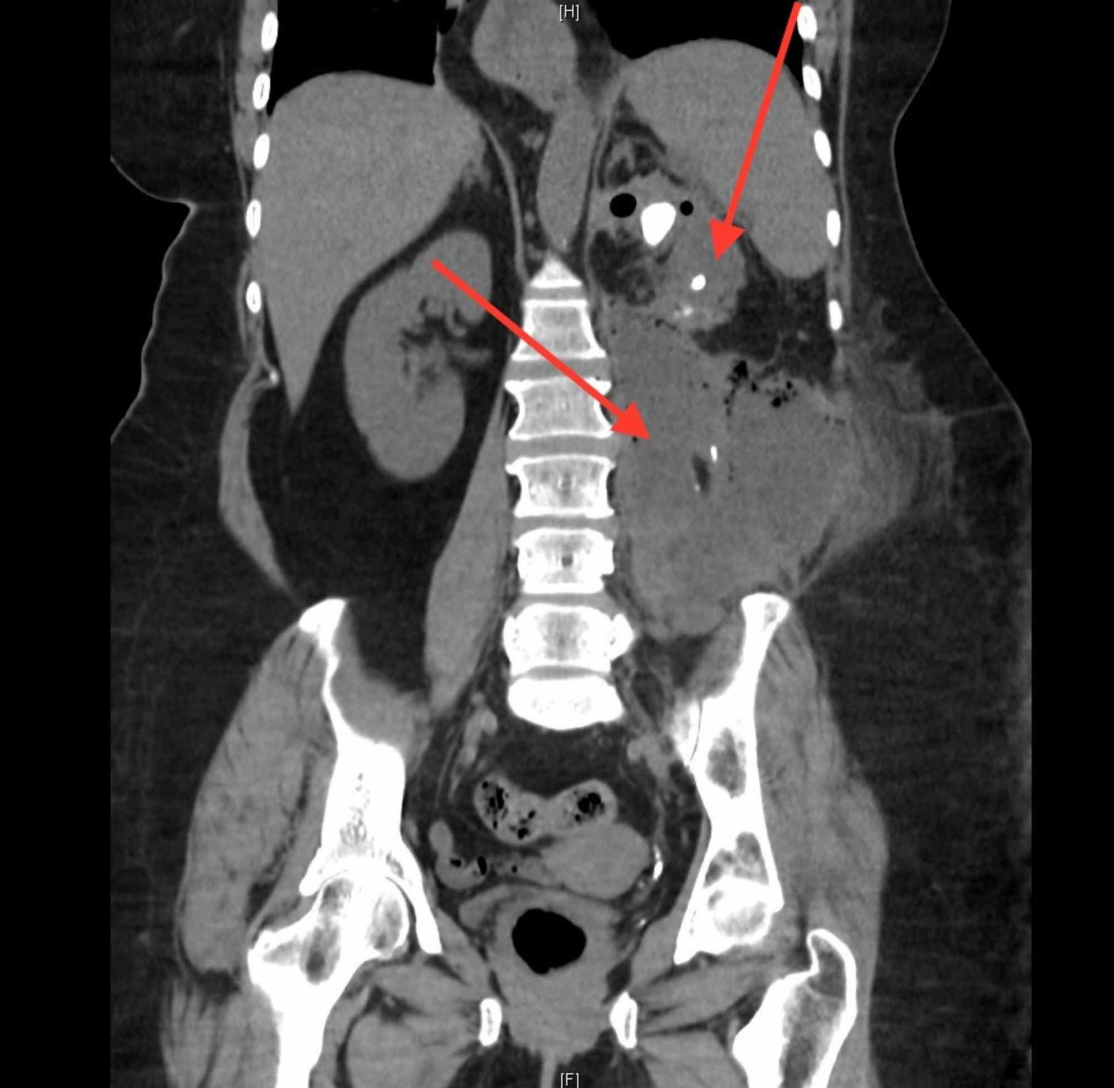
Non-contrast CT: Xanthogranulomatous emphysematous pyelonephritis w/large abscess The top arrow identifies nephrolithiasis and emphysematous pyelonephritis. The bottom arrow identifies the associated perinephric abscess with extension into the pelvic cavity.

A urinalysis revealed pyuria, with a white blood cell count of 477/high power field. Serum testing was remarkable for a leukocytosis of 15,400 cells/µl and creatinine of 3.4 mg/dL. Her baseline renal function was unknown. A non-contrast computed tomography (CT) of her abdomen and pelvis revealed emphysematous pyelonephritis. Also noted were multiple renal calculi within the left ureter and renal pelvis, the largest measuring 15 mm in diameter. Associated with the renal inflammation was a left retroperitoneal abscess, measuring 13.3 x 9.4 x 18 cm in size, extending inferiorly into the pelvis, paraspinal musculature, psoas muscle and fat plane of the left flank (Figure 1). Blood and urine cultures did not yield any organism growth. HIV screening test was negative.

**Figure 2:**
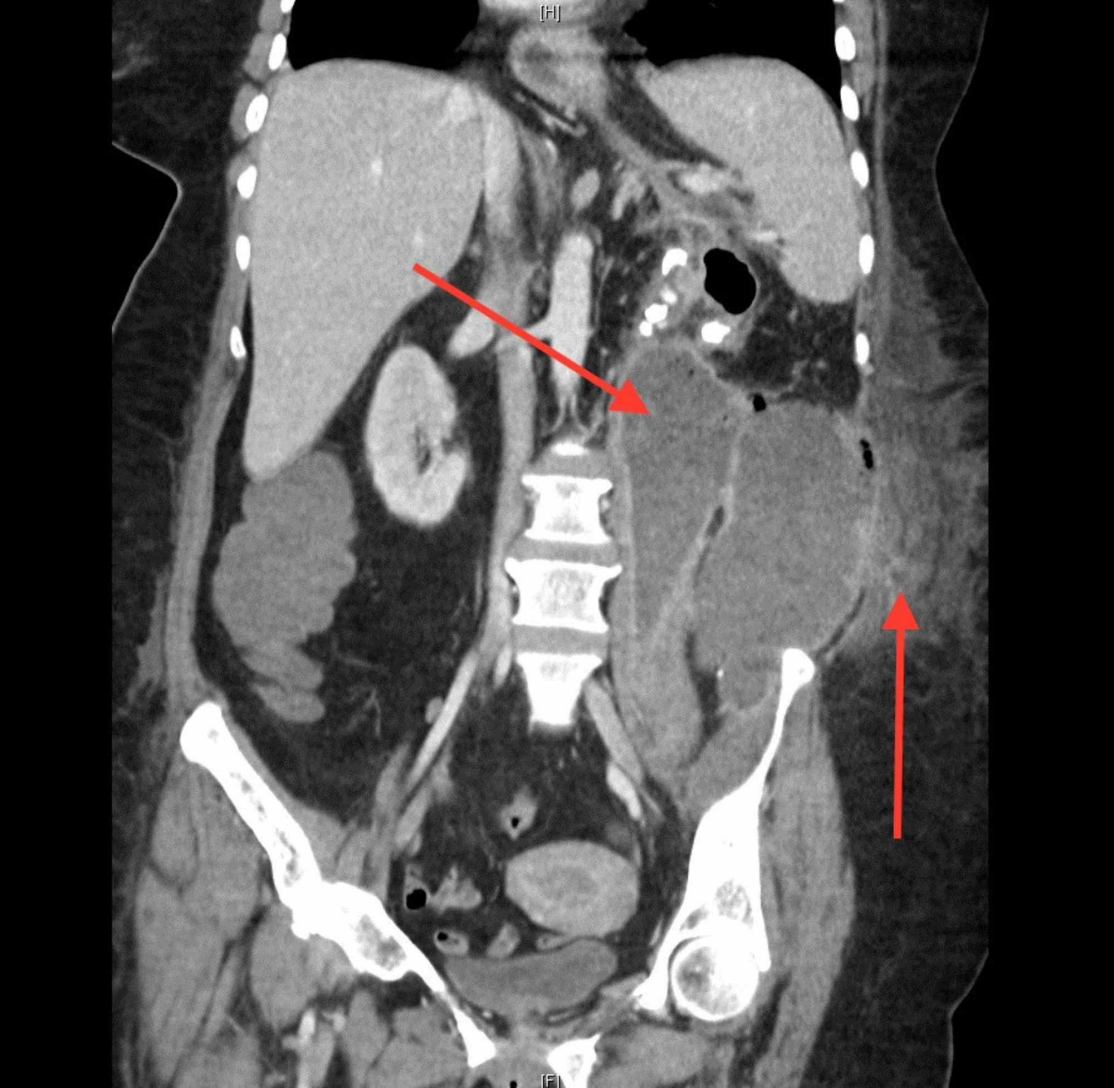
Contrast-infused CT: Subcutaneous extension of abscess with septation The left arrow re-identifies the enlarging, and now septated, perinephric abscess with extension into the pelvic cavity. The right arrow identifies infiltration of abscess into the subcutaneous tissue external to the peritoneal cavity.

Two emergent CT-guided percutaneous tubes were placed with initiation of parenteral vancomycin and imipenem. 250 ml of purulent fluid was drained, with fluid cultures growing pan-sensitive Escherichia coli and Streptococcus anginosus. Fungal and acid-fast bacilli (AFB) smears were negative. Antibiotics were de-escalated to ceftriaxone and metronidazole according to microbial susceptibility. With fluid resuscitation, the serum creatinine downtrended to 1.1 mg/dl by the third hospital day. During the next two days the leukocytosis increased to 25,000/µl, drain output was minimal and the left flank was progressively more swollen and painful, now accompanied by crepitus. A repeat contrast-infused CT revealed an enlarging multiloculated abscess with air-fluid levels and extension into the subcutaneous tissue (Figure 2). There was no mention of vascular invasion, though it was noted radiographically that architecture of surrounding structures, vasculature included, was distorted due to external compression from the abscess. Operative intervention was planned with a two-step approach: abscess drainage with washout, followed by left nephrectomy.

During the first operative phase, 1200 mL of purulent fluid was evacuated and antibiotics were changed to piperacillin-tazobactam as fluid cultures additionally grew Pseudomonas aeruginosa. Post-operatively, the patient’s blood pressure was labile, requiring vasopressor support and red blood cell transfusion. She was presumptively treated for post-operative sepsis secondary to the abscess. On hospital day 11, the patient was taken back to the operating room for definitive nephrectomy. 800 mL pus was drained during the second operation. Mycotic aneurysms of the aorta, celiac and splenic arteries were identified accompanied by massive intraoperative bleeding. Ultimately the splenic artery bleeding could not be controlled and the patient expired on the operating table.

## Discussion

Perinephric abscess is an infrequent but often difficult to treat entity. Resulting from perirenal fat necrosis, nearly 75% are local sequelae of urologic infections [3]. Other predisposing factors include diabetes mellitus, pregnancy, and anatomic urinary tract abnormalities - including nephrolithiasis, vesicoureteral reflux, neurogenic bladder, obstructive tumor, and polycystic kidney disease [1-3,6].

Management of perinephric abscess includes empiric antibiotics against Enterobacteriaceae. Drainage, either surgical or percutaneous, is usually needed both diagnostically and therapeutically [8-11]. Concurrent urologic intervention is also warranted for the management of anatomic abnormalities such as obstructing stones or vesicoureteral reflux. Treatment duration with antibiotics is typically two to three weeks, however is largely dependent upon size and response to infection [10,11,13]. Surgical intervention is indicated with failure of medical therapy and abscesses unlikely to respond to medical therapy alone (i.e., large size) [2,11]. Complicated pyelonephritis, particularly xanthogranulomatous emphysematous pyelonephritis, is also an indication for surgery, particularly nephrectomy for source control.

Untreated abscesses may lead to sepsis and death. More uncommonly, subphrenic abscess, empyema, fistula formation, bowel involvement and peritonitis are reported [2,6,13-15]. Mycotic aneurysms from adjacent non-surgery related inflammation are rarely reported in the literature [12,16].

Symptoms of mycotic aneurysms are non-specific in nature, but may be identified by bacteremia. Definitive diagnosis is made via angiographic methods, including contrast-infused CT [17,18]. Mycotic aneurysm rupture is associated with high rates of morbidity and mortality due to proclivity of rapid expansion and rupture [19,20]. Overall mortality ranges from 10 to 25% for splenic artery aneurysms, and is considerably higher for other sites [20]. Prompt identification and excisional surgery is the cornerstone of therapy [18].

## Conclusions

Perinephric abscess can cause severe illness, especially if extensive in size. Though uncommon, complications from adjacent inflammation such as mycotic aneurysms should be considered when the size of the abscess is massive in nature. Prompt radiographic identification with appropriate imaging is necessary to appropriately manage this rare condition.
